# Structural insights into the mechanism of action of a biparatopic anti-HER2 antibody

**DOI:** 10.1074/jbc.M117.818013

**Published:** 2018-04-18

**Authors:** Vaheh Oganesyan, Li Peng, Jared S. Bee, John Li, Samuel R. Perry, Frank Comer, Linda Xu, Kimberly Cook, Kannaki Senthil, Lori Clarke, Kim Rosenthal, Changshou Gao, Melissa Damschroder, Herren Wu, William Dall'Acqua

**Affiliations:** From the Departments of ‡ Antibody Discovery and Protein Engineering,; § Analytical Sciences, and; ¶ Biosuperiors, MedImmune, Gaithersburg, Maryland 20878

**Keywords:** analytical ultracentrifugation, antibody, crystallography, epitope mapping, phosphorylation

## Abstract

Pathways of human epidermal growth factor (EGF) receptors are activated upon ligand-dependent or -independent homo- or heterodimerization and their subsequent transphosphorylation. Overexpression of these receptors positively correlates with transphosphorylation rates and increased tumor growth rates. MEDI4276, an anti-human epidermal growth factor receptor 2 (HER2) biparatopic antibody–drug conjugate, has two paratopes within each antibody arm. One, 39S, is aiming at the HER2 site involved in receptor dimerization and the second, single chain fragment (scFv), mimicking trastuzumab. Here we present the cocrystal structure of the 39S Fab–HER2 complex and, along with biophysical and functional assays, determine the corresponding epitope of MEDI4276 and its underlying mechanism of action. Our results reveal that MEDI4276's uniqueness is based first on the ability of its 39S paratope to block HER2 homo- or heterodimerization and second on its ability to cluster the receptors on the surface of receptor-overexpressing cells.

## Introduction

The family of epidermal growth factor receptors consists of four members (HER1–4; 1), each of which contains an extracellular domain (comprised of four subdomains), a single-span transmembrane helix, and an intracellular kinase domain that can interact with signaling molecules. All four members of the family are essential mediators of cell proliferation and differentiation (2). They become active through homo- or heterodimerization promoted by ligand binding (3–5). HER2 is the only promiscuous member of the family that can dimerize with any of the other family members and has no need for ligand binding (because it is always in a ligand-bound “open” conformation; 6). Dimerization results in the phosphorylation of tyrosine residues on the intracellular kinase domain, which, in turn, initiates a variety of signaling pathways. Aberrant behavior of cells manifested as overexpression, and activation of HER2 was found to be tightly associated with 20–30% of human breast cancer cases (7, 8). Hence, HER2 has been validated as a therapeutic biomarker and as a target for cancer therapy nearly two decades ago. Inhibition of HER2 activity by using mAb, antibody–drug conjugate (ADC),^7^ or small-molecule kinase inhibitor approaches is frequently used as a therapy for treating HER2-positive metastatic breast cancer. Although these HER2-targeting therapies have improved the overall survival rate, there are still many more cases refractory to these treatments. Among those is a large population of patients whose tumor cells exhibit low HER2 expression levels. Those are clinically categorized as “HER2-negative” despite having HER2 at levels higher than in normal tissues and are not qualified for currently available treatments. Hence, there is a high unmet medical need for developing the next generation of anti-HER2 agents that can target and kill cancer cells with a broad range of HER2 expression levels (9, 10).

**Figure 1.**
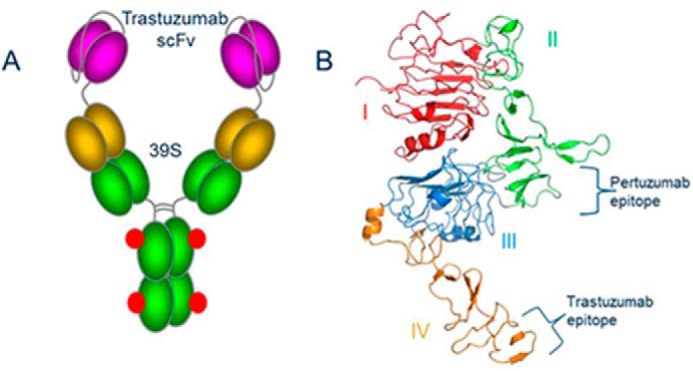
*A*, cartoon of MEDI4276 ADC. Trastuzumab scFv is shown in *magenta*. Linkers between variable heavy and light, scFv and 39S, and the antibody hinge region are shown as *gray coils*. The 39S variable region is shown in *dark yellow*. The 39S constant region is shown in *green*. Conjugation sites are shown as *red dots. B*, structure and domain organization of the HER2 ecd. Subdomains I through IV are colored *red*, *green*, *blue*, and *orange*, respectively. Pertuzumab and trastuzumab epitopes are shown with *curly brackets*. All structure-related figures were prepared using PyMOL (Schrödinger LLC).

Recently, an anti-HER2, biparatopic, monospecific, tetravalent ADC, MEDI4276, was developed to treat a broad range of HER2-expressing metastatic breast cancers (11). It has demonstrated antitumor efficacy in models that are refractory or ineligible to trastuzumab, pertuzumab, or T-DM1 treatment and shows promise as an effective therapy for cancer patient populations not qualified for the abovementioned treatments (11). MEDI4276 was constructed by attaching the single-chain variable fragment (scFv) of trastuzumab to the N terminus of the heavy chain of the anti-HER2 fully human mAb 39S (IgG1κ) (11; Fig. 1*A*). Trastuzumab and 39S bind to nonoverlapping distant epitopes on HER2. Thus, MEDI4276 contains four antigen-binding sites, two on each arm, that are capable of interacting with HER2 (Fig. 1*B*). Such a biparatopic, tetravalent, monospecific antibody was shown to induce robust receptor clustering, internalization, lysosomal trafficking, and subsequent degradation of the complex (11). The presence of four high-affinity binding sites leads to efficient recruitment and clustering of HER2 molecules even in low HER2–expressing tumor models (11). Additionally, to prevent HER2-independent Fcγ receptor–mediated antibody uptake, the L234F substitution in the lower hinge region of the antibody was introduced. This mutation minimizes the probability of toxin molecules entering normal tissues through regular antibody-dependent cellular cytotoxicity mechanisms, thereby reducing off-target cytotoxicity.

The specific property that makes MEDI4276 interact with HER2 more efficiently than regular antibodies and promote receptor clustering remains unknown. In an effort to understand the underlying mechanism of action of MEDI4276, we characterized its binding to HER2 using multiple approaches. We mapped the 39S functional epitope using mutagenesis. We also characterized the MEDI4276–HER2 complexes using multisignal sedimentation velocity (MSSV) analytical ultracentrifugation (AUC). Finally, we determined the X-ray crystal structure of the HER2 extracellular domain bound by 39S Fab. These studies suggest a coherent mechanism of action for MEDI4276.

## Results

### Identification of the 39S epitope

**Figure 2.**
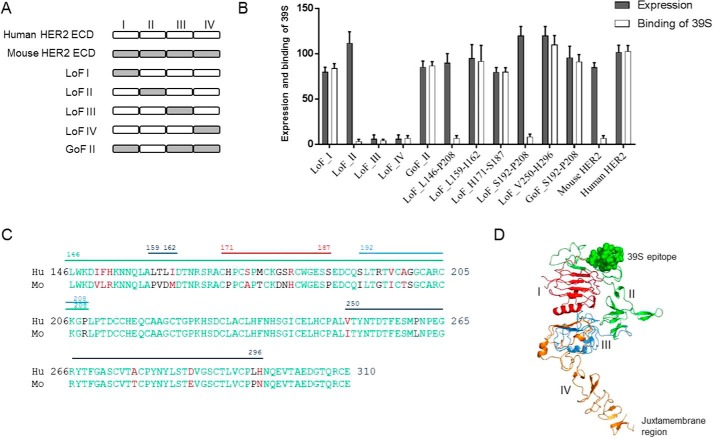
*A*, schematic of the human/mouse chimeric variants. The individual subdomains of the human and mouse HER2 ecd were swapped as illustrated to construct LoF and GoF variants. *B*, binding characterization of 39S to human/mouse chimeric variants. Binding and expression were calculated as percent to WT HER2, dividing the surface plasmon resonance response of 39S or anti-His polyclonal antibody response by that of WT HER2, respectively. Replacing subdomain II or segment Leu^146^-Pro^208^ or Ser^192^-Pro^208^ of human HER2 with mouse counterparts abolished the binding of 39S (LoF II, LoF Leu^146^-Pro^208^, and LoF Ser^192^-Pro^208^). Grafting either subdomain II or the region of Ser^192^-Pro^208^ of human HER2 into the mouse molecule led to recognition of the chimera by 39S (GoF II and GoF Ser^192^-Pro^208^). Results represent the means of three independent experiments, with *error bars* indicating standard deviations. *C*, amino acid alignment of the subdomains II between human (*Hu*) and mouse (*Mo*) HER2. Identical and similar amino acids are shown in *green* and *red*, respectively. Segments denoted Leu^146^-Pro^208^, Leu^159^-Ile^162^, His^171^-Ser^187^, Ser^192^-Pro^208^, or Val^250^-His^296^ with *lines* are ones swapped between human and mouse HER2 to construct chimeric variants. *D*, mapping the 39S functional epitope on HER2 structure. The functional epitope of 39S, shown as *spheres*, is the most distant from the juxtamembrane part of the molecule.

To map the epitope of 39S at a domain level, we constructed five chimeric human/mouse HER2 variants by swapping in or out their subdomains (I, II, III, or IV) within the extracellular domain (ecd) (Fig. 2*A*). Mouse HER2 was selected as the chimeric partner because it shares 85% amino acid sequence identity with human HER2 but is not recognized by 39S. The chimeric variants were constructed and expressed as soluble proteins with a His_6_ tag using human embryonic kidney (HEK) 293F cells. Protein expression and binding of 39S to the variants were assessed using surface plasmon resonance by capturing the variants on the sensor surface using an anti-human HER2 polyclonal antibody (Fig. S1). Three of the five chimeric variants were well expressed, as monitored with an anti-His antibody. Two loss-of-function (LoF) variants had no detectable expression when replacing subdomains III and IV (LoF III and LoF IV). Antibody 39S did not recognize the LoF variant, in which subdomain II was replaced with its mouse counterpart (LoF II) (Fig. 2*B*). Moreover, 39S bound well to the gain-of-function (GoF) variant, in which the human HER2 domain II was grafted into mouse HER2 (GoF II). We also found that the binding affinities of 39S to the GoF II variant and the entire HER2 ecd were comparable (data not shown), suggesting that the other subdomains of HER2 ecd (I, III, and IV) are unlikely to be involved in significant interaction with 39S. Therefore, domain II of HER2 is likely to be the sole contributor to 39S binding. To determine the exact site of 39S binding, short peptide segments in subdomain II of human and mouse HER2 were swapped. Five chimeric variants were generated by replacing the following human HER2 regions with the corresponding mouse counterparts: Leu^146^-Pro^208^, Leu^159^-Ile^162^, His^171^-Ser^187^, Ser^192^-Pro^208^, or Val^250^-His^296^ (Fig. 2*C*). Protein expression and binding of 39S to these variants were characterized as described above using surface plasmon resonance. All variants expressed at levels comparable with that of WT HER2. Antibody 39S bound to most variants but did not recognize those encoding for the mouse region of Ser^192^-Pro^208^ (Leu^146^-Pro^208^ and Ser^192^-Pro^208^) (Fig. 2*B*). However, 39S did bind to the GoF Ser^192^-Pro^208^ variant made by introducing the segment of human Ser^192^-Pro^208^ HER2 into the mouse molecule. These results suggest that the functional epitope of 39S spans amino acids Ser^192^-Pro^208^ of subdomain II and consists of a short, double disulfide–constrained loop. This area of HER2 is one of the most distant places from the membrane-proximal subdomain IV, where trastuzumab scFv binds (Fig. 2*D*).

### Crystal structure of the 39S Fab–HER2 complex

**Figure 3.**
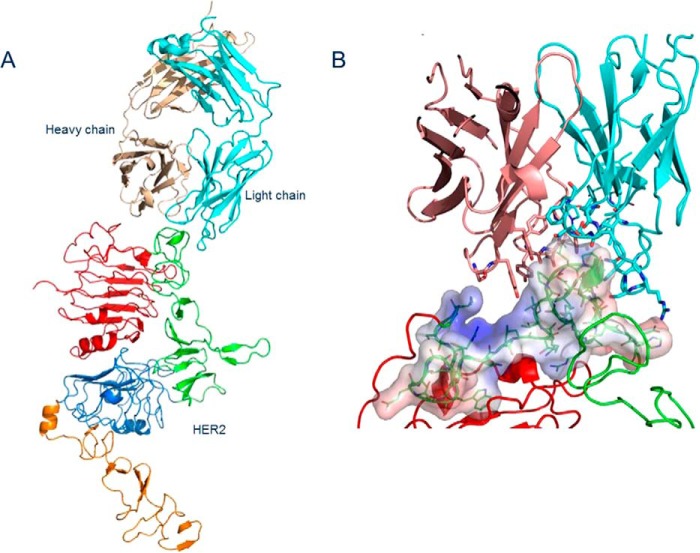

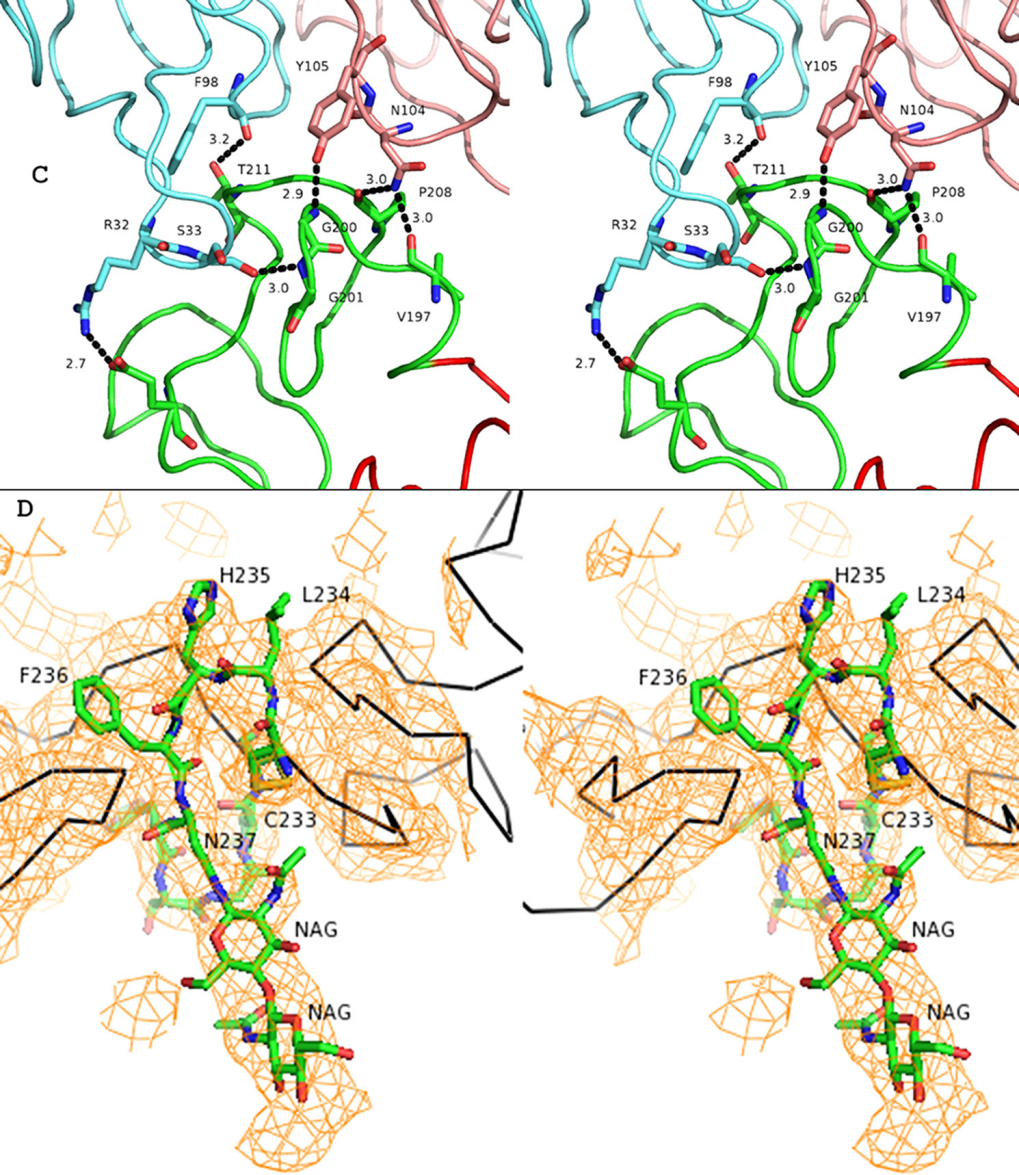
*A*, content of an asymmetric unit of crystal containing 39S Fab and HER2 ecd. HER2 domains I through IV are colored *red*, *green*, *blue*, and *orange*, respectively. Fab heavy and light chains are shown in *sand* and *cyan*, respectively. *B*, view of interaction between 39S Fab and HER2 domain II. Electrostatic surface potential, calculated for the tip of domain II using the APBS plugin in PyMOL, shows that nature of the interaction is mostly hydrophobic. *C*, close-up view showing the interaction of both chains of 39S Fab with domain II of HER2. The coloring scheme is as in *A* and *B. D*, fragment of the final electron density map around glycosylated Asn^237^ showing two consecutive GlcNAc residues at 1.6 σ. The carving radius is 9 Å. The structure was determined to a resolution of 3.8 Å.

The crystal structure of the complex formed by the extracellular domain of HER2 and 39S Fab was determined by molecular replacement, using the structure of human HER2 extracellular domain (PDB code 3BE1, 12) and an unrelated unpublished Fab structure as search models. There is one 39S Fab–HER2 complex in the asymmetric part of the unit cell (Fig. 3*A* and Table 1). The conformational arrangement of HER2 subdomains is virtually identical to that determined in other studies of HER2 alone or in complex with trastuzumab and pertuzumab Fabs. The conformation has been assessed by superpositioning structures and visually reviewing their alignment. The classification and conformational arrangement of antibody CDR loops was examined using PyIgClassify (13), an online canonical structure classification tool according to North *et al.* (14). 39S Fab binds to an epitope that includes the end of subdomain I and the beginning of subdomain II of HER2, spanning amino acids 197 to 216. That epitope is positioned in the shallow grove formed between CDRs H2 (H2–10^−2^ CDR–length combination) of heavy and L1 (L1-17-1) of light chains (Fig. 3*B*). Upon HER2 binding, about 660 Å^2^ of solvent-accessible area is sequestered. Three hydrogen bonds are detected between the heavy chain of 39S and HER2, all coming from CDR3 (H3-14-4). These include H3:Asn^104^ Nδ2–A:Pro^208^ O, H3:104N Nδ2–A:Val^197^ O, and H3:Tyr^105^ Oη–A:Gly^200^ N. Five hydrogen bonds are contributed by CDR1 and 3 (L3-9-cis7-1) of the light chain and include both side chain and main chain interactions (L1:Arg^32^ Nη1–A:Glu^216^ Oϵ1; L1:Ser^33^ Oγ–A:Gly^201^ N; L3:Phe^98^ O–A:Thr^211^ Oγ1; L3:Phe^98^ O–A:Thr^211^ N). CDRs H1, H2, and L2 do not participate in antigen binding directly (Fig. 3*C*). However, hydrophobic residues also contribute to a substantial portion of the interaction of both chains of 39S with HER2. The electron density map covers amino acids 1 to 575, leaving the last 55 residues invisible. Carbohydrates at glycosylated asparagine positions A:Asn^46^, A:Asn^165^, and A:Asn^237^ have a well-defined electron density for two initial GlcNAc residues (Fig. 3*D*).

**Table 1:** **X-ray data and model refinement statistics** Values in parentheses correspond to the highest-resolution shell.

Wavelength (Å)	1.0000
Resolution (Å)	40–3.77 (3.77–3.87)
Space group	P2_1_2_1_2_1_
Unit-cell parameters (Å, °)	a = 67.33, b = 108.17, c = 348.31
Total reflections	301,349
Unique reflections	25,241
Completeness (%)	99.94 (95.0)
*R*_sym_	0.13 (0.87)
Mean *I*/σ(*I*)	12.0 (1.0)
Mosaicity (°)	0.8
Multiplicity	12.4 (10.6)
Resolution (Å)	40–3.77
*R*_work_/*R*_free_/*R*_work+free_	0.251/0.289/0.253
RMSD bonds (Å)	0.01
RMSD angles (°)	1.43
Number of protein atoms	7,819
Mean B-factor (model/Wilson) (Å^2^)	77.2/63.5

### Multisignal sedimentation velocity analytical ultracentrifugation

**Figure 4.**
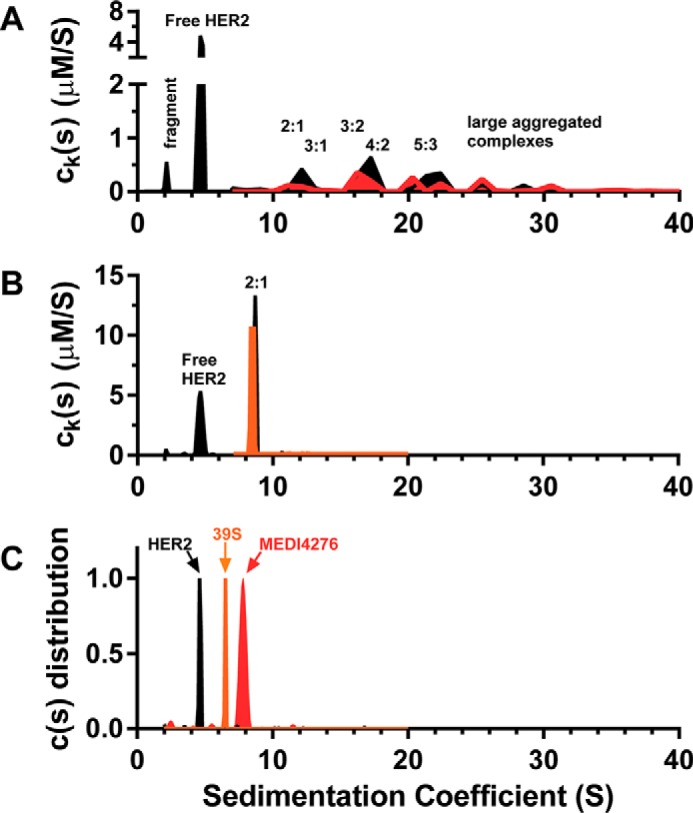
*A* and *B*, MSSV results for 4:1 loading of the HER2 and MEDI4276 biparatopic antibody (*A*) and 4:1 loading of HER2 and the 39S mAb (*B*). *C*, control samples of HER2, 39S mAb, and MEDI4276, which were run separately and plotted together with a normalized *y* axis. HER2 is depicted as a *black fill*, 39S mAb in *orange*, and MEDI4276 in *red*.

AUC experiments were performed using different ratios of soluble HER2 (∼86 kDa), MEDI4276 (∼208 kDa), and 39S (∼148 kDa) molecules. Fig. 4 shows the sedimentation coefficient distributions from the MSSV experiment for a 4:1 loading ratio of HER2 and MEDI4276 biparatopic (Fig. 4*A*), a 4:1 loading ratio of HER2 and the comparator 39S mAb (Fig. 4*B*), and control samples of HER2, 39S mAb, and MEDI4276, which were run separately and plotted together (Fig. *4**C*). The MSSV analysis gives a separate c(s) profile specific for each protein based on its unique spectral “fingerprint.” Therefore, if the protein is present both free and within one or more complexes, then multiple peaks will be detected at s values corresponding to masses of the complexes. The quantity of that protein within each of the complexes is equal to the area under its curve. Therefore, the ratio of the areas of each protein within each peak can be used to determine their ratio in the complex. The MSSV peak area analysis confirmed that HER2 formed a single 2:1 complex with the 39S mAb with a sedimentation coefficient of 8.6S (Fig. 4*B*). The formation of a complex limited to 2:1 stoichiometry is the expected behavior for a regular antibody–antigen interaction such as 39S.

In contrast, rather than a single saturated complex, MEDI4276 biparatopic assembled together with HER2 into a variety of complexes over the range of 10S to 30S. The fact that peaks of complexes were broad suggests that the sample contained a heterogeneous mixture of HER2–MEDI4276 complexes assembled into configurations with different shapes and frictional ratios that may not be fully resolved experimentally. The experimentally determined sedimentation coefficient was used to estimate the mass of the complex using the Svedberg equation (15). Using the solution properties for phosphate-buffered saline and a universal protein partial specific volume of 0.73 ml/g, the relationship can be expressed as S (calculated) ≅ (0.0035M^2/3^)/(f/f_0_).

In calculations, we used the experimentally determined frictional ratio f/f_0_ of 1.78. The mass of the peak from its sedimentation coefficient was used together with the ratio of species determined from the MSSV analysis to assess the stoichiometry of the complexes present in the solution (Fig. 4*A*).

The broad peak detected from 10S to 13S is consistent with a molecular mass of between about 364 kDa and 540 kDa (consistent broadly with complexes ranging from 2:1 to 4:1). The average ratio of HER2 to MEDI4276 was 2.2:1 in this peak. These MSSV data are consistent with a complex with a stoichiometry of 2:1 (expected mass = 380 kDa, S = 10) with likely some smaller quantity of unresolved 3:1 (expected M = 466 kDa, S = 12) complexes. The expected sedimentation coefficient of a “saturated” 4:1 complex would be about 13S, which is higher than the experimentally observed value of 2.2:1 for this peak.

The broad peak detected from 15.5S to 18S is consistent with a molecular mass between 703 kDa and 880 kDa. The average ratio of HER2 to MEDI4276 was 1.7:1 in this peak. These results suggest that there are incompletely resolved complexes with a 3:2 (expected M = 675 kDa, S = 15) and 4:2 (expected M = 761 kDa, S = 16) stoichiometry in this region. The width of the peak and the differences between the experimental data and the prediction may reflect variation of different shapes and frictional ratios of unresolved complexes.

The broad peak detected from 20S to 22S is consistent with molecular masses of the order of 1 MDa. The average ratio of HER2 to MEDI4276 was also 1.7:1 in this peak. Complexes with a stoichiometry of 5:3 (expected mass = 1.1 MDa, S = 20.3) with some possibly unresolved contribution from 3:3 and 4:3 is consistent with this region. It is also likely that there are different configurations and shapes of formed complexes with different frictional ratios, resulting in increased heterogeneity of the sample and, subsequently, in broadening of the observed peaks.

The region at S > 22 contained a ratio of 0.5 HER2 to each MEDI4276 on average. However, the potential stoichiometry of the larger species is likely to be less reliable based on the lower signal and high heterogeneity of the sample and might be best interpreted as “larger aggregated complexes.” Based on their sedimentation values, the complexes in the region of 24S to 26S are expected to have a mass of about 1.5 MDa, and the complexes in the region of 28S to 32S are expected to have a mass of between 1.7 and 2.1 MDa.

In prior studies, HER2–MEDI4276 complexes were characterized using size exclusion chromatography with multiangle light scattering detection (SEC-MALS) (11). The estimated masses of the complexes ranged from 500 kDa to 1.7 MDa, depending on the ratio of HER2 to MEDI4276 (11). Complexes eluted as broad unresolved peaks, and some eluted in the void volume of the SEC column. SEC-MALS has potential for changes to the complexes because of column interactions or higher shear flow. The light-scattering signal intensity is biased to larger species, which may also limit the ability to characterize unresolved smaller complexes.

The current AUC results confirm the formation of large HER2–MEDI4276 complexes. A greater separation and resolution of smaller complexes into discrete peaks was achieved by AUC. The distribution of complexes formed was found to be dependent on the ratio of HER2–MEDI4276 in both the prior study (11) and this study and was different in both studies. The MSSV approach allowed for estimation of the ratio of species in each complex, allowing the stoichiometry to be established. Therefore, when considering these differences in the SEC-MALS and AUC techniques, the identification of complexes by AUC is expected to be of higher resolution. Overall, the results by AUC are in good agreement and confirm the prior SEC-MALS results.

The MSSV analysis has confirmed that, dependent on the availability of receptors, MEDI4276 assembled with HER2 to form various complexes with HER2:MEDI4276 stoichiometric ratios of major species ranging from 2:1 to 5:3. Exclusive formation of 4:1 stoichiometric complexes was not observed. However, they may be present in solution as minor species. The presence of major peaks consistent with, among other species, 2:1, 3:2, and 5:3 stoichiometric complexes might reflect an overall energetic and steric favoring of cross-linking of complexes to form larger aggregated species rather than complete limited filling of each MEDI4276 biparatopic molecule with either two or four HER2 antigens.

### Inhibition of ligand-induced Akt phosphorylation

**Figure 5.**
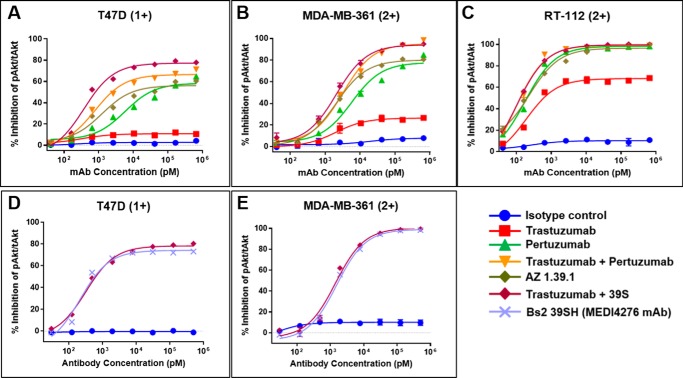
*A–C*, inhibition of Heregulin 1-β1–dependent Akt phosphorylation was evaluated in the breast cancer T47D (*A*), MDA-MB-361 (*B*), and bladder cancer RT-112 (*C*) cell lines using a multiplexed Ser(P)^473^/total Akt sandwich immunoassay kit. *D* and *E*, the similarity of inhibition of HRG1-β1–dependent Akt phosphorylation by unconjugated MEDI4276 (*Bs2–39SH*) and combination of the parental antibodies. HER2 expression was evaluated in tumor xenografts by IHC test (data not shown), and the IHC intensity scoring is shown in *parentheses* for each cell line.

HER2-targeted antibodies trastuzumab and pertuzumab (Herceptin® and Perjeta®, respectively) inhibit HER2-mediated signaling by distinct but complementary mechanisms (16, 17, 18). Pertuzumab directly blocks the receptor dimerization necessary for ligand-dependent signaling, whereas trastuzumab inhibits the mitogen-activated protein kinase and phosphatidylinositol 3-kinase/Akt pathways by a complex mechanism that is still not fully understood (19). We examined the effect of 39S, alone or in combination with trastuzumab, on the ligand-dependent phosphorylation of Akt. The breast cancer cell lines T47D and MDA-MB-361 and the bladder cancer cell line RT112 were selected based on immunohistochemistry staining for HER2 on tumor xenografts using the HercepTest^TM^ IHC kit (data not shown). These models demonstrated HercepTest^TM^ IHC scores of 1+ (T47D) or 2+ (RT112 and MDA-MB-361), representing tumors that would be ineligible for currently available HER2-targeted therapies. As shown in Fig. 5, trastuzumab exhibited modest inhibition of Akt phosphorylation in these cell lines, as shown by a multiplexed Ser(P)^473^/total Akt immunoassay (MesoScale Discovery). 39S inhibited Akt phosphorylation more potently than trastuzumab, whereas the combination of 39S and trastuzumab produced the greatest degree of inhibition. The enhanced effect of the combination on inhibition of Akt phosphorylation was most pronounced in the HER2 IHC cell line RT-112. For comparison, we tested the ability of the HER2 dimer–blocking antibody pertuzumab alone or in combination with trastuzumab. As a single agent, pertuzumab-mediated inhibition of Akt phosphorylation was comparable with that of 39S. The combination of pertuzumab and trastuzumab elicited more potent inhibition, but it was not superior to the trastuzumab and 39S combination. These results suggest that 39S and trastuzumab have complementary activity with respect to inhibition of the phosphatidylinositol 3-kinase/Akt signaling pathway. The unconjugated version of MEDI4276 (Bs2–39SH) inhibited Akt phosphorylation with equal potency compared with the combination of the two parental antibodies, as shown in Fig. 5, *D* and *E*. The result of this experiment suggests that combining the two antibodies into a biparatopic mAb did not impair the ability to block HRG1-β1–dependent downstream signaling.

## Discussion

**Figure 6.**
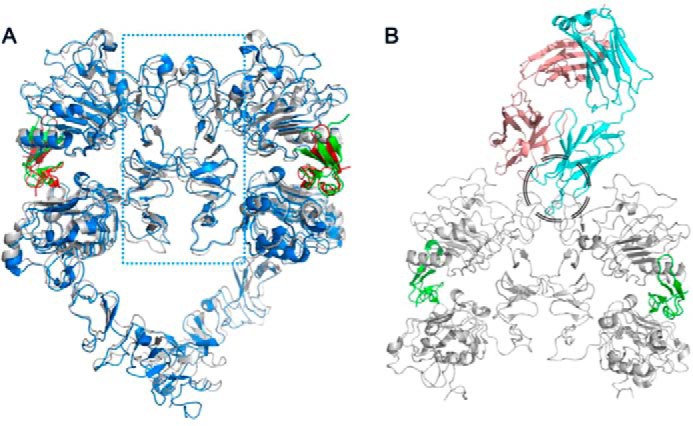
*A*, crystal structures of HER1 (*blue* and *red* for HER1 and EGF, respectively; PDB code 3NJP; 36) and HER4 (*gray* and *green* for HER4 and neuregulin, respectively; PDB code 3U7U; 22) ligand-induced dimers, superimposed, show that the dimerization interface is solely made of subdomains II (the area within the *blue dotted rectangle*). *B*, superimposition of either of those homodimers with the 39S Fab-HER2 structure shows that 39S causes steric hindrance and prevents homo- and heterodimerization of HER molecules. The area of clash is shown by the *black circle*.

The 135-kDa transmembrane glycoprotein HER2 differs from other members of its family by not having a cognate ligand. Instead, it readily heterodimerizes with three other family members and induces signaling. A prerequisite for this heterodimerization is the presence of the ligand in other receptors. It is worth noting that, at higher expression levels, HER2 receptors can also heterodimerize in a ligand-independent manner (20). Because all four members of this family exhibit very similar domain organization and share ∼40% sequence similarity, homodimerization could also be expected. Indeed, epidermal growth factor–induced homodimers of HER1 and neuregulin-induced homodimers of HER4 have been observed (21, 22). Hetero- and homodimerization interfaces of HERs are believed to be similar and solely made of subdomain II (Fig. 6*A*), the very subdomain that contains the 39S epitope. Superimposition of the HER2–39S Fab complex structure with structures of HER1 and/or HER4 homodimers allowed us to confirm that the presence of 39S Fab prevents formation of both homo- and heterodimers (Fig. 6*B*). This, in turn, suggests that no phosphorylation should be detected in the presence of either 39S IgG or MEDI4276. This was confirmed by results of AKT phosphorylation experiments (Fig. 5). Thus, we postulate that the mechanism of action of mAb 39S relies on prevention of HER2 homo- or heterodimer formation.

**Figure 7.**
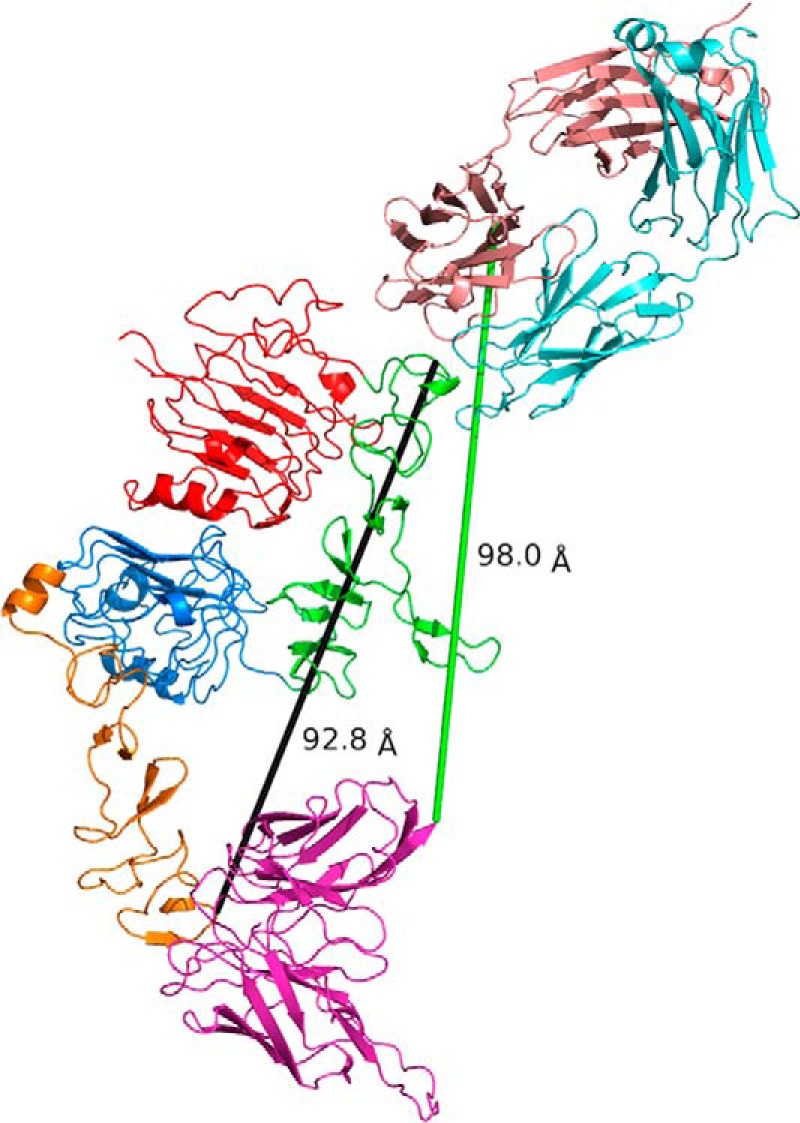
39S and trastuzumab epitopes are located at opposite ends of HER2 molecule at a distance of more than 90 Å from each other (*black line*). The (G_4_S)_3_ linker employed in MEDI4276 can hardly stretch to even 60 Å and will not be able to cover the distance of 98 Å (*green line*) that would be required to allow the C-terminal residue of trastuzumab scFv and N-terminal amino acid of 39S heavy chain to bind simultaneously to the same antigen molecule. This suggests that MEDI4276 cannot bind two HER2 receptors and have all its paratopes occupied at the same time.

The biparatopic antibody format employed in MEDI4276 uses trastuzumab scFv upstream of the heavy chain of 39S IgG (Fig. 1*A*). Each arm of this antibody can theoretically bind either one antigen molecule at two different sites (provided the linker between scFv and Fab is long enough to cover the distance between the two corresponding epitopes) or two separate antigen molecules. The linker between the C-terminal residue of trastuzumab scFv and the N-terminal residue of the 39S heavy chain in MEDI4276 consists of three G4S repeats. G4S repeats are very common and convenient linkers, allowing production of fusion proteins in a single chain and providing some space between functional units (23). They are also known for not being structured (24). One such repeat, in its most stretched conformation, may allow molecules on both ends to be ∼20 Å apart. Accordingly, three of those repeats can theoretically be stretched to ∼60 Å. This falls short of the distance that would be required to allow the C-terminal residue of trastuzumab scFv and N-terminal amino acid of the 39S heavy chain to bind simultaneously to the same antigen molecule (∼ 100 Å, Fig. 7). It is also worth noting that, in practice, the hydrodynamic radius of IgG molecules containing one through nine G4S linkers in the hinge is independent of the number of repeats (24). Hence, we conclude that one arm of MEDI4276 will not be able to bind concurrently both epitopes on the same HER2 molecule and will have to bind two antigen molecules to saturate all of its binding sites. This is in agreement with our AUC experimental results. The absence of masses corresponding to two antigens bound to one antibody at antigen:antibody ratios above 3:1 supports our conclusion of one arm not being able to bind one antigen with both paratopes.

Biparatopic antibodies constitute a particular case of bispecific molecules that recognize two different epitopes on the same antigen. Currently there are over 100 different bispecific antibody formats in the public domain (34), and all of them have been developed for specific properties. Which format will ultimately exhibit the desired properties will very much depend on the physical and biological properties of the antigen, the corresponding mechanism of action (*e.g.* agonist *versus* antagonist), and, most importantly, the developability of the chosen format. In the case of MEDI4276, the biparatopic format allowed for the combination of several essential properties: HER2 functional dimerization can be effectively blocked by the 39S arm, the distance between trastuzumab scFv and 39S Fab epitopes is greater than the distance between those paratopes within each arm of MEDI4276, and the close proximity of several HER2 molecules triggers their internalization and efficient degradation. In summary, the corresponding epitope of MEDI4276 and its underlying mechanism of action determined by the cocrystal structure of 39S Fab–HER2 and biophysical and functional analyses can also be used to inform the design of next-generation antibody therapeutics for improved potency. Increased receptor binding and cross-linking of a bivalent biparatopic antibody targeting two nonoverlapping epitopes can induce robust internalization, lysosomal trafficking, and degradation of ADCs to release an active drug, therefore improving the therapeutic efficacy. Similarly, a biparatopic bispecific antibody targeting a soluble antigen can form large immune complexes that can bind Fcγ receptors on phagocytic cells and are rapidly internalized for fast antigen clearance, thus decreasing the serum level of the targeted antigen (35). Moreover, a biparatopic antibody with the ability to not only neutralize but also actively drive clearance of soluble antigens could lead to further improvements in therapeutic efficacy.

## Experimental procedures

All chemicals employed were of analytical grade. MEDI4276 and 39S antibodies were generated as described previously (11, 25). All antibody and antigen amino acid positions mentioned in the text were identified according to a consecutive numbering scheme.

The cell lines MDA-MB-361 and T47D were obtained from the ATCC. The cell line RT112 was obtained from the Deutsche Sammlung von Mikroorganismen und Zellkulturen (Germany). MDA-MB-361 cells were cultured in Lebovitz medium supplemented with 20% fetal bovine serum. T47D and RT112 cells were cultured in RPMI1640 growth medium (Thermo Fisher/Life Technologies) supplemented with 10% heat-inactivated fetal bovine serum. The cells were cultured at 37 °C in a humidified growth chamber at 5% CO_2_ and passaged by subculturing following trypsinization.

DNA encoding chimeric human/mouse soluble HER2 variants with a His tag were assembled and amplified by overlapping PCR using human and mouse HER2 (NCBI references NP_001005862.1 and NP_001003817.1, respectively) plasmids as templates (MedImmune). The assembled DNA was cloned into a mammalian expression vector pEBNA (MedImmune). HEK293F cells were then transiently transfected with these constructs using 293fectin according to the manufacturer's instructions (Invitrogen). Cell culture supernatant was harvested 6 days after transfection for binding characterization using surface plasmon resonance.

The binding characteristics of 39S to chimeric human/mouse soluble variants were investigated using a ProteOn XPR36 instrument (Bio-Rad). Standard amine coupling was used to immobilize an anti-human or mouse HER2 polyclonal antibody (R&D Systems) in 10 mm sodium acetate (pH 5.0) to the surface of a GLC biosensor chip at ∼5000 resonance units for each channel. The chimeric proteins in cell culture supernatant were injected and captured by anti-human or mouse polyclonal antibodies onto the GLC surface with a ∼200-resonance unit response. Antibody 39S was diluted in PBS (pH 7.4) with 0.005% Tween 20 from 10 nm to 0.625 nm (1:2 dilution) and injected at 100 μl/min for 180 s with a 600-s dissociation time. Expression levels of chimeric variants were monitored by flowing anti-His polyclonal antibody (MedImmune) under the same conditions as when injecting 39S. The surface was regenerated twice by injecting glycine buffer (10 mm (pH 1.5)) at flow rate of 100 μl/min for 30 s. All sensorgram data were processed with the ProteOn Manager 3.0.1 software.

MSSV AUC was used to evaluate the stoichiometry of complexes formed between the HER2 antigen and the MEDI4276 antibody. For screening, a series of samples containing HER2 and MEDI4276 at molar ratios of 1:2, 1:1, 2:1, and 4:1 in PBS buffer were analyzed by AUC sedimentation velocity. Sedimentation coefficient distributions, c(s), were generated from the raw data using the Sedfit software as described by *Schuck et al.* (26).

The MSSV experiment was performed at a molar loading of antigen to MEDI4276 ratio of 4 to 1 (5.8:1.4 μm) based on the screening results, showing that there was excess free HER2 in addition to formation of a variety of large complexes, as shown in Fig. S1. Identical datasets were collected for mAb 39S, an mAb limited to bivalent binding, for comparison. Samples and reference buffer were loaded into 12-mm double-sector cells with Epon centerpieces and sapphire windows. The cells were placed in an An50-Ti rotor mounted in a Beckman Optima XL-I centrifuge set to 20 °C. Data scans were collected at 42,000 rpm with a radial resolution of 0.002 cm over the range of 5.9–7.2 cm. Signals were acquired from UV at 280 and 250 nm and from the interference detection system. Global multisignal analysis using the program SEDPHAT was used to determine the stoichiometry of the complexes by taking advantage of the differences in the signal increments of the individual proteins according to the procedure described by Balbo *et al.* (27). Control samples of HER2 antigen, 39S mAb, and MEDI4276 bispecific were used to determine the signal increments of each species for interference and UV detection at 250 nm using the known UV extinction values at 280 nm. The density and viscosity of PBS were estimated using the Sednterp program (28). The partial molar volume was set to 0.73 g/ml for the control 39S mAb and MEDI4276 bispecific. To account for the glycosylation of HER2 (17 kDa of 86 kDa), the partial molar volume was set to 0.71 g/ml for analysis of samples containing HER2. The frictional ratios and meniscus positions were allowed to float. A single segment over the range 2S to 20S with a resolution of 0.1S was used for the MSSV data analysis of the control single-species samples. Segments were defined to allow the fitting procedure to adjust the frictional ratio of the species detected at different sedimentation coefficient ranges. Two segments at resolution of 0.1S were used for the 4:1 sample of HER2 and 39S mAb. Segment 1 was defined over the range of 2S to 7S and segment 2 from 7.1S to 20S. Two segments were used to fit the data for the 4:1 sample of HER2 and MEDI4276 bispecific. Segment 1 was defined to contain only free HER2 from 0.5S to 6.9S at a resolution of 0.2S. Segment 2 was defined to contain both HER2 and MEDI4276 bispecific from 7.0S to 50S at a resolution of 1S.

The ability of HER2 antibodies to inhibit HRG1-β1–dependent Akt phosphorylation was evaluated using a multiplexed Ser(P)^473^/total Akt electrochemiluminescent sandwich immunoassay kit (MesoScale Diagnostics). The breast cancer cell lines T47D and MDA-MB-361 and the bladder cancer cell line RT-112 were used in this study. Cells were disaggregated by trypsinization and washed with PBS once to remove serum. Cells were resuspended in assay medium (RPMI 1640 with 0.1% BSA, serum-free) at 5.0 × 105 cell/ml. 100 μl of cells (50,000 cells/well) were seeded into opaque-walled 96-well plates and incubated overnight in a 37 °C incubator. The following day, the cells were treated for 60 min at 37 °C with a 4-fold dilution series of HER2 or control mAbs in assay medium as indicated. Cells were then stimulated with HRG1-β1 (R&D Systems) at a final concentration of 1 μm. After 15 min at 37 °C, the assay medium was decanted, and cells were lysed with complete lysis buffer containing protease and phosphatase inhibitor mixtures according to the manufacturer's recommendations. The resulting cell lysates were transferred to a clean U-bottomed 96-well plate and centrifuged to pellet any cellular debris. 25 μl of clarified lysate from each well was transferred to MesoScale Diagnostics Multi-Spot Ser(P)^473^/total Akt assay plates, and assay procedures were carried out according to the manufacturer's recommendations. The plates were read on a Sector Imager 6000 instrument, and the data were captured automatically using the instrument software and analyzed using Excel.

The Fab portion of 39S IgG1 was prepared by digesting the mAb with papain, passing the digestion mixture over a Mab Select Sure column (GE Healthcare), and collecting the Fab in flow-through. His tag–bearing HER2 was recombinantly expressed in HEK293F cells and purified over an immobilized metal affinity chromatography (IMAC) affinity column (GE Healthcare) and a size exclusion Superdex200 (GE Healthcare) column (11). The complex was formed by mixing nearly equimolar amounts of HER2 and Fab with a slight excess of Fab to achieve full saturation of HER2, followed by complex purification over a Superdex200 (GE Healthcare) column. Diffraction quality crystals of HER2–39S Fab were grown in hanging drop format by mixing equal volumes of protein complex stock solution of 9.6 mg/ml with solution containing 10% w/v PEG8000, 20% v/v ethylene glycol, and 0.02 m carboxylic acid mixture in 0.1 m MES/imidazole buffer (pH 6.5) at ambient temperature. Upon harvesting, crystals were flash-cooled in liquid nitrogen. X-ray diffraction data from a single crystal were collected at a wavelength of 1000 Å and a temperature of 100 K at the X06DA Beamline of the Swiss Light Source equipped with a Pilatus 2M detector. Three hundred sixty frames were recorded using an oscillation range of 0.5°, a crystal to detector distance of 400 mm, and an exposure time of 0.25 s. X-ray diffraction data were processed with the HKL2000 program (29). The structure was solved using the program Molrep (30) and refined in the CCP4 suite (31) using REFMAC5 (32). The model was manually adjusted using “O” (33).

## Author contributions

V. O. designed the experiments, performed crystallization of the complex, collected X-ray diffraction data, solved and refined the structure, wrote the manuscript, and prepared illustrations. L. P. designed and performed knock-in and knock-out mutational studies and helped with writing the first draft of the manuscript. J. S. B. performed and interpreted analytical ultracentrifugation experiments and wrote the corresponding pages of the manuscript. J. L. and S. R. P. performed phosphorylation experiments. F. C. wrote the portion of the manuscript describing phosphorylation experiments and prepared the associated illustrations. L. X., K. C., K. S., L. C., and K. R. provided support for the expression and purification of proteins. C. G., M. D., and H. W. critically read and commented on the manuscript. W. D. helped with writing the final version of the manuscript.

## Supplementary Material

Supporting Information

## Footnotes

7 The abbreviations used are:

ADC: antibody–drug conjugate
scFv: single chain fragment variable
MSSV: multisignal sedimentation velocity
AUC: analytical ultracentrifugation
ecd: extracellular domain
HEK: human embryonic kidney
LoF: loss-of-function
GoF: gain-of-function
SEC-MALS: size exclusion chromatography with multiangle light scattering detection
IHC: immunohistochemistry
CDR: complementarity-determining region.
