# Supplementary material for: Structural insights into the mechanism of action of a biparatopic anti-HER2 antibody
Source: J Biol Chem. 2018 Apr 18;293(22):8439–48. doi: 10.1074/jbc.M117.818013 (PMC5986207; doi:10.1074/jbc.M117.818013)
Supplement: Supporting Information [file supp_293_22_8439__index.html]

Structural insights into the mechanism of action of a biparatopic anti-HER2 antibody. — Structural insights into the mechanism of action of a biparatopic anti-HER2 antibody — Biparatopic antibody binding to the HER2 receptor — Supporting Information 

# Structural insights into the mechanism of action of a biparatopic anti-HER2 antibody

## Supporting Information

- Supplemental figure 1 (.docx, 136 KB) - Supplemental figure 1
