# Supplementary material for: Structural insights into the mechanism of action of a biparatopic anti-HER2 antibody
Source: J Biol Chem. 2018 Apr 18;293(22):8439–48. doi: 10.1074/jbc.M117.818013 (PMC5986207; doi:10.1074/jbc.M117.818013)
Supplement: Supporting Information [file 10.1074_M117.818013_jbc.M117.818013-1.docx]

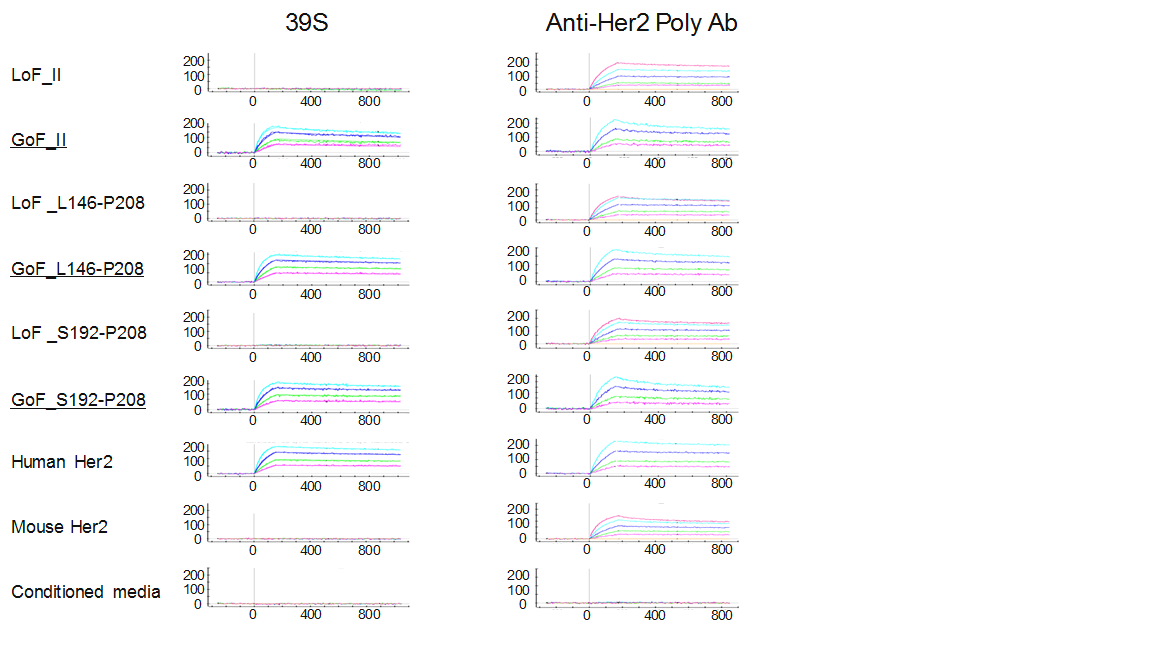


Supplemental Figure 1. The surface plasmon resonance sensorgrams (raw data) of the binding and expression characterization of a set of variants discussed are presented. Chimeric molecules generated by swapping corresponding segments of human and mouse HER2 ecd were folded properly and were stable as shown by polyclonal Ab binding data and by gain-of-function experiments.
